# Membrane Bound Monomer of Staphylococcal α-Hemolysin Induces Caspase Activation and Apoptotic Cell Death despite Initiation of Membrane Repair Pathway

**DOI:** 10.1371/journal.pone.0006293

**Published:** 2009-07-21

**Authors:** Saumya S. Srivastava, Satyabrata Pany, Amita Sneh, Neesar Ahmed, Aejazur Rahman, Krishnasastry V. Musti

**Affiliations:** National Centre for Cell Science, University of Pune Campus, Pune, India; Universidade Federal do Rio de Janeiro (UFRJ), Instituto de Biofísica da UFRJ, Brazil

## Abstract

**Background:**

Wild type Staphylococcal α-hemolysin (α-HL) assembly on target mammalian cells usually results in necrotic form of cell death; however, caspase activation also occurs. The pathways of caspase activation due to binding/partial assembly by α-HL are unknown till date.

**Results:**

Cells treated with H35N (a mutant of α-HL that remains as membrane bound monomer), have been shown to accumulate hypodiploid nuclei, activate caspases and induce intrinsic mitochondrial apoptotic pathway. We have earlier shown that the binding and assembly of α-HL requires functional form of Caveolin-1 which is an integral part of caveolae. In this report, we show that the caveolae of mammalian cells, which undergo a continuous cycle of ‘kiss and run’ dynamics with the plasma membrane, have become immobile upon the binding of the monomer. The cells treated with H35N were unable to recover despite activation of membrane repair mechanism involving caspase-1 dependent activation of sterol regulatory element binding protein-1.

**Conclusions:**

This is for the first time we show the range of cellular changes and responses that take place immediately after the binding of the monomeric form of staphylococcal α-hemolysin.

## Introduction

Binding of pore forming toxins such as staphylococcal α-HL can cause significant changes in cellular signaling of many cell types [1], [2]. The water soluble form of this protein binds to the target cell as a monomer which then recruits other such monomers (which have undergone conformational changes in a similar fashion) to form a non-lytic, pre-pore assembly. This pre-pore assembly then undergoes further conformational changes to form a heptameric, mushroom like transmembrane pore to destabilize the membranes [3]. Formation of transmembrane pores on the target cells result in osmotic imbalance of the cell leading to death by necrotic pathway. α-HL's assembly on Jurkat cells resulted in necrotic form of cell death even though caspases were found to be active [4], [5]. An intriguing question that remained unanswered as to how the caspases are activated by α-HL's assembly? The answer probably lies in the nature of the structural form of α-HL that is present on the cell surface as the presence of functional pore on the target cell membrane is anticipated to result in osmotic imbalance and necrotic form of cell death. Theoretically, when α-HL binds to the target cell, all the three forms, *viz.*, the cell bound monomer, the non-lytic pre-pore and the lytic pore can be present as only a fraction of the bound α-HL undergoes all the conformational changes to form the lytic pore. The precise nature of disturbances in the cellular signaling, caused by the other two non functional forms, i.e. the membrane bound monomer and the non-lytic pre-pore forms of α-HL are still not clear. In order to understand the changes in cellular signaling due to the membrane bound monomeric form of α-HL, we have employed a mutant, *viz.*, H35N (Histidine 35 mutated to Aspargine) that cannot assemble beyond membrane bound monomer although it folds like the wild type protein. The Histidine-35 of α-HL is essential for efficient inter-protomer interactions during the oligomerization [6]. In the present study, we demonstrate that the H35N arrests the dynamics of caveolae at the cell surface, initiates the membrane repair pathway. Probably due to absence of adequate repair response, the target cells induced apoptosis via the intrinsic mitochondrial pathway.

## Materials and Methods

Annexin-V-FITC apoptosis detection kit was obtained from Calbiochem. FITC conjugated active anti-Casapse-3 monoclonal antibody detection kit was obtained from BD-Pharmingen. Propidium iodide, Hoechest 33342, LDH kit and trypan blue were obtained from Sigma Chemical Co., USA. Cav-1(pY14) mouse monoclonal antibody, Anti-PARP(cleaved) antibody were procured from BD Biosciences. Phospho p38, SREBP-1, caspase-1, anti-Actin antibody and zVADfmk were from Santacruz Biotech. p38, Caveolin-1, caspase-3, cytochrome C antibody and all the corresponding HRP conjugated secondary antibodies were bought from Cell Signaling Technologies. JC-1 dye was obtained from Molecular Probes. Fluorescein Active caspase-9 staining kit was purchased from Biovision. Lipofectamine 2000 was purchased from Invitrogen.

### Purification of H35N and α-HL

The mutant of α-HL in which the Histidine 35 of α-HL replaced with Aspargine (viz. H35N) was constructed as described earlier [6]. α-HL and H35N were cloned and expressed in *E. coli* JM109(DE3) under the control of T7 promoter and purified as described earlier [7], [8].

### Cell culture and toxin treatment

A431 and HeLa cells were cultured in DMEM medium containing 10% FCS in the presence of Penicillin-G and Streptomycin sulfate. Cells at approximately 60–80% confluency were treated with the H35N (8 µg/ml) or α-HL (800 ng/ml) in the complete DMEM unless specified otherwise.

### Morphological studies on A431 cells

Monodispersed cells after 10–12 hr of plating were incubated with H35N (8 µg/ml) or α-HL (800 ng/ml) in complete media for 10 hr and photographed under light microscopy.

### Immunoblot analysis

Cells were incubated with α-HL (800 ng/ml) or H35N (8 µg/ml) for the desired time at 37°C, following which adherent and floating cells were recovered and washed with cold PBS (pH 7.4). The cell pellet was resuspended in lysis buffer (150 mM NaCl, 1% NP-40, 1 mM EDTA, 1 mM PMSF, 2 mM Sodium orthovanadate and protease inhibitor cocktail) and the supernatant was passed through 20 gauge syringe for 15–20 times, nuclei and cell debris were pelleted at 14,000 g for 20 min. The supernatant was estimated for protein amount and electrophoresed on 12% SDS-PAGE for Cav-1, p38, caspase-3 and Caspase-1and 15% SDS-PAGE for cytochrome C. For PARP proteolysis 10% SDS-PAGE gel was used.

### Flow cytometric detection of apoptotic cells by Propidiumiodide staining

For the hypodiploid nuclei, cells after treatment with the toxins for the mentioned time, were harvested by trypsinisation and washed twice in cold PBS followed by fixation with chilled 70% ethanol for 1 hr and spun at ∼1500 g for 5 min. The cell pellet was washed twice with cold PBS and treated with RNaseA (50 µg/ml) for 30min at 37°C. The cells were chilled on ice for 10 min and stained with Propidium Iodide (50 µg/ml) for 1 hr and analysed by FACS vantage flow cytometer and 10,000 events were measured for each sample. For the analysis of morphological changes, cells were treated with H35N or pre-treated with zVADfmk for 2 hr followed by H35N treatment and stained with PI (2 µg/ml) for 5 min. Live cells were analyzed by FACS.

### Flow cytometric detection of active caspase-3 and Caspase-9

Cells incubated with toxin for different time points, harvested by mild trypsinisation, followed cold PBS wash and finally resuspended in cytofix/cytoperm solution (part of FITC conjugated active anti caspase-3 antibody monoclonal antibody detection kit purchased from BD Pharmingen) and incubated on ice for 20 min. The cells were washed twice with perm wash buffer at room temperature, the antibody was added as per the manufacturer's protocol and incubated for 30 min at room temperature followed by washing once with perm wash buffer and resuspended in 0.4 ml of wash buffer and analyzed by flow cytometry. Activation of caspase-9 was determined using caspglow fluoroscein caspase-9 staining kit (Biovision). Cells were harvested by mild typsinisation and washed with PBS. According to the manufacturer's protocol FITC-LEHD-FMK was added to these cells and incubated for 45 min at 37°C followed by washing twice with PBS. The cells were resuspended in 300 µl of wash buffer and analyzed by flow cytometry using FL-1 green channel and 10,000 events were measured for each sample.

### Annexin-V-Propidium iodide dual staining

Floating and adhered cells were harvested together by mild trypsinisation after toxin treatment, washed twice with PBS and incubated with FITC conjugated annexin-V (0.5 µl/1×10^4^ cells) and PI (10 µg/ml). In each sample, dual parameter dot plot of red versus green fluorescence signal was obtained and at least 10,000 events were measured.

### Detection of the mitochondrial membrane potential (ΔΨm)

The mitochondrial membrane potential was analyzed using the JC-1 dye. Both floating and adherent A431 cells after treatment were harvested together by mild trypsinisation and washed twice with cold PBS. The pellet was resuspended in PBS containing JC-1 (7 µg/ml) and incubated in dark at 37°C for 20 min. The resultant cells were washed twice with PBS, resuspended in 0.4 ml PBS and analyzed by Becton Dickinson FACS system. A minimum of 10,000 cells per sample were analyzed.

### Immunofluorescence analysis

For Hoechst staining, cells were treated with H35N or α-HL or pretreated with zVADfmk (50 µM) for 2 hr followed by H35N treatment for the indicated time points. The cells were fixed with Paraformaldehyde (3.7%) for 10 min at room temperature followed by washing with PBS (pH 7.4). The cells were stained with 1 µM Hoechst 33342 in PBS for 15 min at 37°C. The unbound stain was removed by washing twice with PBS. For SREBP-1 staining, the cells were fixed with 3.7% paraformaldehyde for 10 min at room temperature, permeabilised with 0.1% NP-40 for 5 min. Blocked with 3% BSA and labeled with anti SREBP-1 monoclonal antibody followed by Cy-2 conjugated secondary antibody. The cover slips were scanned using Zeiss Laser scanning microscopy. The nuclei were stained with DAPI.

### Trypan blue staining

A431 or HeLa cells were left untreated or treated with H35N (8 µg/ml) or pretreated with zVADfmk (50 µM) for 2 hr followed by addition of H35N (8 µg/ml) for the indicated time points and the cells were mildly trypsinised for few minutes following inactivation of trypsin in complete media. The cells were thoroughly washed with PBS. Equal numbers of cells were stained with trypan blue (0.2%) for 2 min and stained cells and total cells were counted on hemocytometer.

### Subcellular fractionation

Cytosolic lysates were prepared in a buffer containing 250 mM sucrose, 20 mM HEPES-KOH (pH 7.4), 10 mM KCl, 1.5 mM Na EGTA, 1.5 mM EDTA, 1 mM Magnesium Chloride, 1 mm DTT and protease inhibitor cocktail. Cells were kept in the above buffer for 30 min at 4°C followed by homogenization at 4°C and spun at 10,000 g at 4°C. The supernatant was further spun at 18,000 g for 30 min at 4°C to yield cytosolic extract. Cytosolic extract (60 µg) was electrophoresed on 15% SDS-PAGE. Membrane and nuclear extract were prepared as described by Wang et al [9]. All operations were carried out at 4°C. Briefly cells were harvested, washed twice with PBS and centrifuged at 1800 g for 10 min, pellets were suspended in buffer A (10 mM HEPES-KOH at pH 7.6, 1.5 mM Mgcl_2_, 10 mM KCl, 0.5 m DTT, 1 mM EDTA, 1 mM EGTA) supplemented with protease inhibitor, disrupted by dounce homogenization and centrifuged at 1000 g for 10 min. The resulting crude nuclear pellet was extracted with buffer B (20 mM HEPES-KOH at pH 7.6, 25% {v/v} glycerol, 0.5 mM Nacl, 1.5 mM Mgcl_2_, 1 mM EDTA, 1 mM EGTA) supplemented with protease inhibitor and centrifuged either at 25,000 g for 30 min. The supernatant was designated as nuclear extract. The supernatant from the initial low speed centrifugation (above) was spun at 100,000 g for 1 hr. The membrane fraction was washed with buffer B by centrifugation at 100,000 g to obtain a washed membrane pellet. The pellet was resuspended in 1% (w/v) SDS contaning 10 mM Tris-HCl pH 6.8, 100 mM NaCl. After incubation for 15 min at room temperature, the mixtures were centrifuged at 100,000 g for 30 min at 15°C. The supernatant was designated as membrane fraction. 80 µg of each fractions were analysed by SDS- PAGE followed by Western blotting using SREBP-1 monoclonal antibody.

### LDH release assay

A431 cells were treated in serum free medium for the given time points and then the media was removed and spun at 2000 g for 10 min to pellet down cells, if any. The LDH release in the media was assayed using *in vitro* LDH release kit from Sigma by following manufacturer's protocol. The reaction mixture was added in double the volume of the media and was kept in dark for 45 min and then the reaction was stopped by adding 1/10 volume of 1 N HCl to each well. The absorbance was measured at 490 nm.

### TIRF recording of HeLa and A431 cells transiently transfected with Cav-1-GFP

HeLa or A431 cells (2.5×10^5^) were plated in pre-washed cover slips and at 60–80% confluency, the medium was exchanged with fresh, serum free medium without antibiotics. The cells were transfected with Cav-1-GFP DNA (1 µg) using Lipofectamine 2000 (Invitrogen, USA) as per the manufacturer's suggestions. Before 12–14 hr of the TIRF recording, the cells were transferred to phenol red free DMEM medium and just before the recording the cover glass was transferred to an Atto chamber and placed in about 800 µl of the same medium. The cells were examined for background and optimal expression of Cav-1-GFP. We have routinely selected cells that showed sub-optimal to optimal expression of Cav-1-GFP to observe clear, distinct dynamics as shown in the videos (over expression leads to excessive signal at the cell surface which obstructs the observations). Video streams of caveolae dynamics were acquired in an Olympus IX-81 TIRF microscope. The Cav-1-GFP was excited with a 488 nm Ar laser and the emission signal was collected through emission filter specific for GFP and images were recorded with a Cascade 512B camera after adjusting the total internal reflection angle. A 100X, PlanApo, N.A. 1.4, TIRF objective was used for all acquisitions. All the recordings were made using 10 MHz digitizer with EM gain of 3(4X) and exposure time of 50 msec. The laser power was adjusted with neutral density filters present in-line which provided about 20% of the full power. The shutter of the Ar laser was open only during the recording (90-100 frames at 22–24 frames per second) and all other parameters such as laser intensity, EM gain of the camera, exposure time, frame rate and length of recording remained constant throughout the experiment. Video were recorded before treatment and after treatment with H35N (5 µg/ml) or W179C (5 µg/ml) or α-HL (500 ng/ml) for 2–3 hr. Each TIRF video presented in this work is a representative of at least 4–6 independent measurements. The motion data was analyzed by ImageJ and Microsoft Excel together.

## Results and Discussion

Considering the nature of the action of pore forming toxins on mammalian cell membranes, we anticipated that mere binding, without pore formation, should not result in either cell death or substantial changes in the signaling pathways of target cells except initiating steps for repairing the membrane damage, if any. The present study is aimed at understanding the full range of changes that take place and their influence on cellular signaling soon after the binding of the staphylococcal α-HL to target cells. We have carried out the present study with the help of H35N mutant of α-HL, an oligomerization deficient mutant of the toxin. The H35N does not assemble beyond the membrane bound monomeric stage on target cells as it cannot form efficient inter-protomer interactions to form a pre-pore [8]. Earlier studies have shown that low doses of α-HL treatment of Jurkat T cells resulted in caspase activation via mitochondrial pathway and oligonucleosomal DNA fragmentation. Furthermore, the cell death was not inhibited by zVADfmk or overexpression of Bcl-2 and the process was concluded to be independent of death receptor pathway [4].

### α-HL and H35N are cytotoxic to the target cells

**Figure 1:**
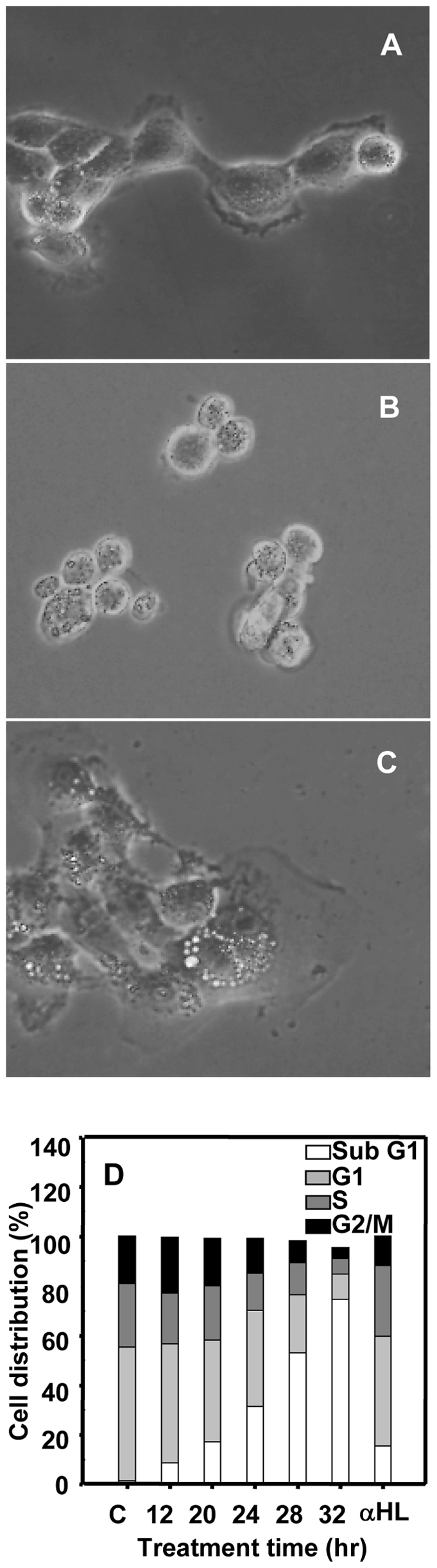
H35N induces morphological changes and increase in sub G1 population in A431 cells: Cells were left untreated (A) or treated with H35N for 10 hr (B) or α-HL for 10 hr (C). After 10 hr, the cells were viewed using phase contrast microscope (D) A431 Cells were treated with H35N or α-HL for the indicated time points and stained with propidium iodide as mentioned in material and methods and hypodiploid nuclei was analyzed by flow cytometry.

In the present study, the morphology of A431 cells after H35N treatment showed an extensive rounding and cell shrinkage as compared to the untreated cells (Fig. 1A, B & C). We have also observed that both α-HL and H35N were found to be cytotoxic to A431 and HeLa cells in a dose dependent manner (data not shown). The percentage of cell death caused by H35N was about 65% and that caused by α-HL was about 75% in 30 hr for A431 cells and a similar magnitude of cytotoxicity was also observed for HeLa cells. Moreover, treatment of A431 cells with H35N resulted in accumulation of hypo-diploid DNA and a time dependent increase in the sub-G1 population of cells, while α-HL treatment did not exhibit a significant sub-G1 peak (Fig. 1D), demonstrating that the cell death caused by H35N is mechanistically different from that caused by α-HL.

### H35N mediates Chromatin condensation in A431 and HeLa cells

**Figure 2:**
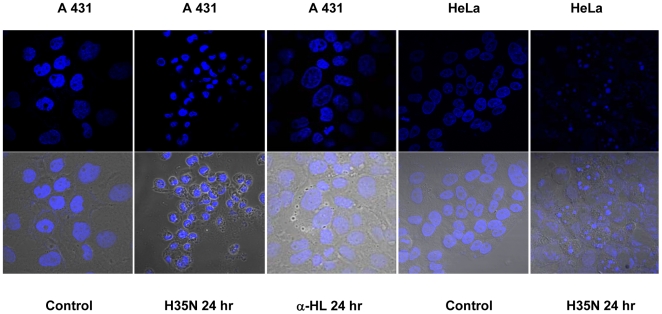
H35N induces condensation of nuclei assessed by Hoechst 3342 staining: A431 and HeLa cells were treated with H35N or α-HL for 24 hr as indicated in the figure. The cells were analyzed using 407 nm laser in confocal microscope. Lower panels show merged image of Hoechst staining with phase contrast.

Chromatin condensation, a marker event of apoptosis, was monitored by Hoechst 33342 staining. A431 or HeLa cells incubated with H35N for 24 hr distinctly showed condensed nuclei in comparison to the untreated cells (Fig. 2). α-HL treated A431 cells did not exhibit any nucleus condensation, demonstrating that H35N induced cell death is mechanistically distinct from the α-HL induced death (Fig. 2).

### H35N activates caspases, induces PARP cleavage

**Figure 3:**
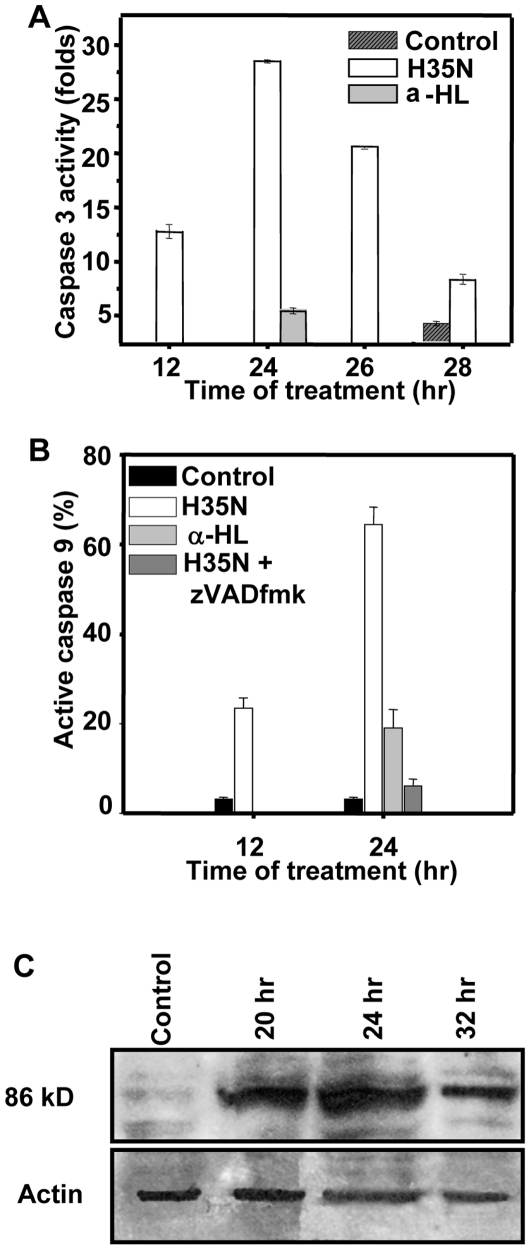
H35N induces caspase-3 activation, caspase-9 activation, PARP cleavage: (A) A431 cells were incubated with H35N, α-HL for the mentioned time and active caspase-3 level was measured by using FITC labeled anti active caspase-3 antibody as mentioned in materials and methods. The graph represents the average of two independent experiments. (B) Following treatment of A431 cells with H35N or α-HL or H35N+zVADfmk for the indicated time points, the cells were processed for flow cytometry as mentioned in materials and methods. The graph is a representation of the average of two independent experiments. (C) A431 cells were left untreated or treated with H35N. The cell lysate was prepared as described in materials and methods and subjected to immunoblotting using PARP antibody which recognizes the cleaved PARP only. The lower panel represents actin as loading control. The position of the molecular weight is indicated on the left.

In view of this unanticipated cell death, we have investigated the nature of cell death in detail. Immunoblotting analysis showed cleaved forms of caspase-3 (17 and 20 kD) appeared after 12 hr of H35N treatment which were consistent with the bands seen in case of Cisplatin treatment (500 µM), a potent apoptosis inducer (data not shown). In contrast, α-HL showed no caspase-3 cleavage. The presence of active caspase-3 was also confirmed by FITC labeled active anti-caspase-3 monoclonal antibody staining in A431 cells. In comparison to the untreated cells, a seven fold increase in the active caspases-3 in H35N treated cells was seen in 24 hr (Fig. 3A) while α-HL showed negligible increase in active caspase-3. In order to elucidate whether or not the mitochondrial pathway was responsible for H35N induced caspase-3 activation, untreated or α-HL or H35N treated A431 cells were examined for the presence of active caspase-9. Similar to caspase-3 activation, H35N induced time dependent increase (∼70% in 24 hr) in FITC-LEHD-FMK staining in A431 cells (Fig. 3B). In contrast, α-HL treated cells showed just ∼25% caspase-9 activity in 24 hr. Also, A431 cells pretreated with zVAD-fmk, a broad spectrum caspase inhibitor, inhibited the caspases-9 activation induced by H35N (Fig. 3B). Caspase-3 activation was further confirmed by PARP proteolysis (Fig. 3C).

### H35N induces mitochondrial pathway of apoptosis

**Figure 4:**
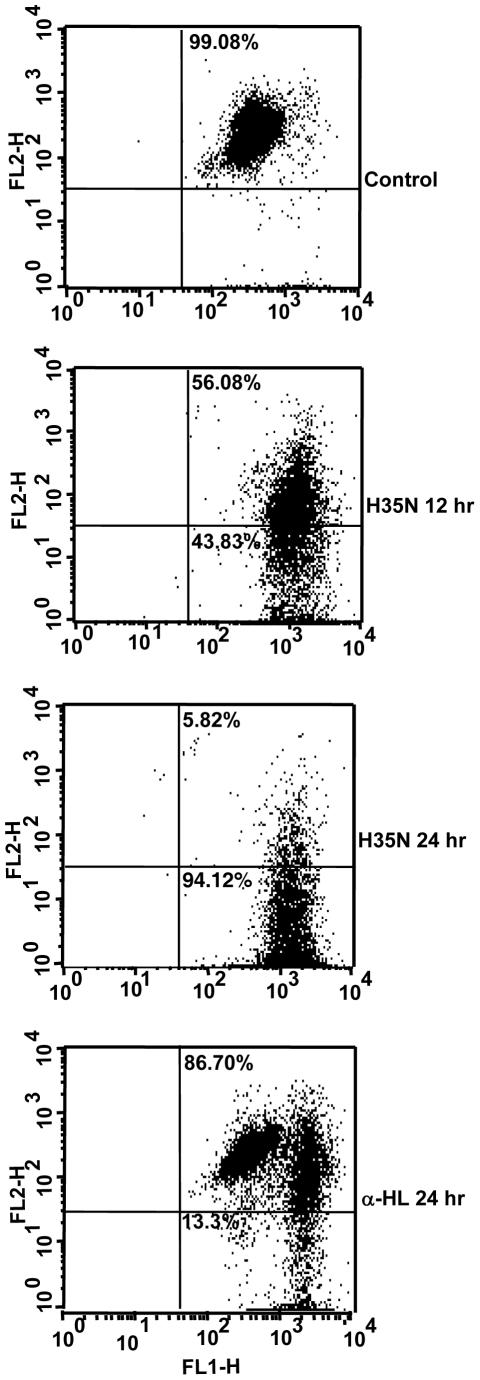
Effect of H35N on mitochondrial membrane potential using JC-1 staining: A431 cells either left untreated or treated with H35N (8 µg/ml) or α-HL (800 ng/ml) for the mentioned time was stained with JC-1 following manufacturer's protocol as mentioned in materials and methods and assessed by flow cytometry. Here FL-1 H signifies green fluorescence whereas FL-2H represents red fluorescence. One representative experiment of the two independent sets of experiments is shown.

**Figure 5:**
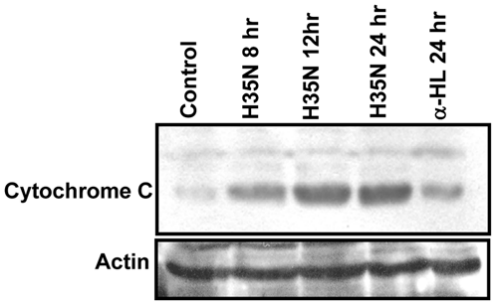
H35N mediates cytochrome C release: H35N induced cytochrome C release assessed by immunoblotting. A431 cytosolic lysate was prepared post treatment with H35N or α-HL for indicated time using protocol mentioned in materials and methods section. Lower panel represents total actin.

To determine whether mitochondria were affected by H35N, we first examined the depolarization of mitochondrial membrane potential by measuring the fluorescence emission shift (red to green) of the ΔΨm sensitive cationic JC-1 dye. JC-1 is readily taken up by mitochondria of healthy cells as a monomer. This uptake increases the concentration of the JC-1 inside the mitochondria leading to the formation JC-1 aggregates which appear greenish red, whereas, depolarized mitochondria do not accumulate JC-1 which remains in the cytoplasm as green colored monomer. Therefore, increase in green to red ratio symbolizes depolarization of mitochondria. H35N treatment showed a time dependent increase in green/red fluorescence intensity of A431 cells loaded with the JC-1 dye (Fig. 4). It is known that cytochrome C release from intermembraneous space of mitochondria into the cytosol is one of the key event in the activation of caspase-9, which subsequently initiates the caspase cascade. Since H35N induces the activation of caspase-9, we also investigated for the cytochrome C release in the cytosolic fraction after H35N treatment in order to define the upstream event in H35N induced apoptosis. Immunoblot analysis clearly showed a time dependent increase in cytochrome C release whereas α-HL showed a marginal increase in cytochrome C release as seen in Fig. 5. The mitochondrial membrane depolarization and release of cytochrome C signify that the apoptotic cell death, induced by H35N, proceeds via an intrinsic pathway. Hence, our observations on apoptotic cell death are consistent with the earlier observations that described apoptotic death of Jurkat T cells by low doses of α-HL [4].

### Caspase inhibition could not prevent H35N induced cell death

**Figure 6:**
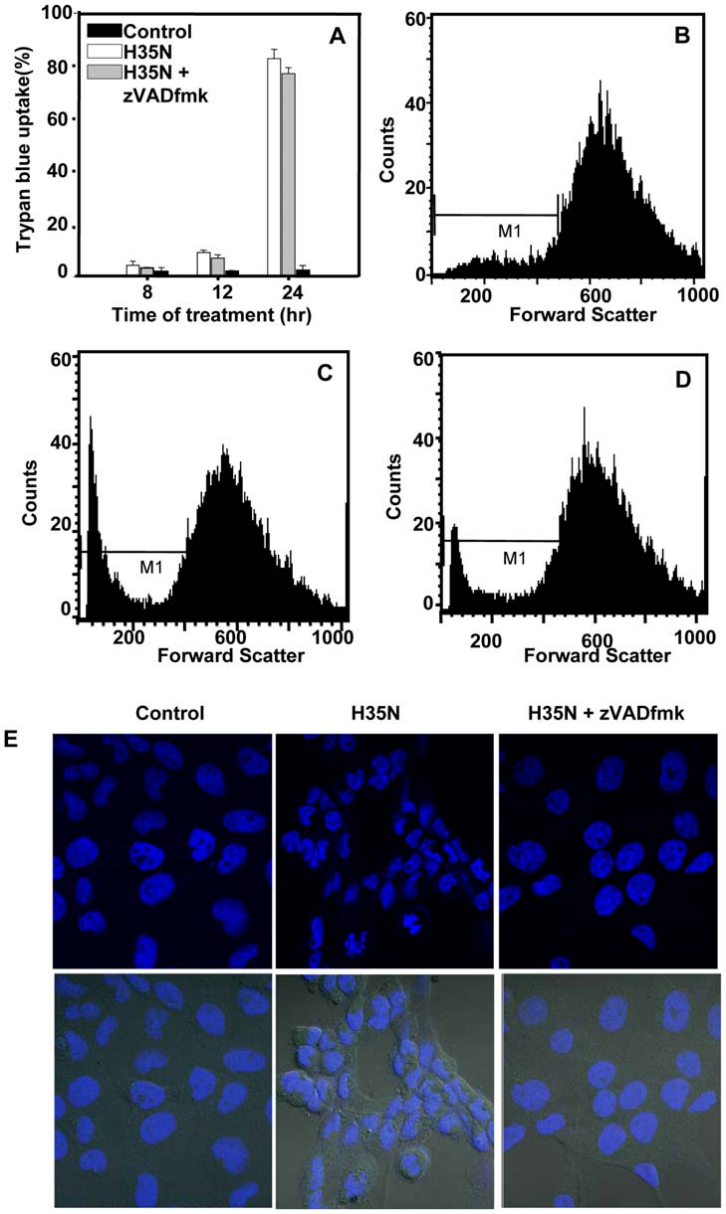
H35N induced cell death and changes in nuclear morphology in presence of pan-caspase inhibitor zVADfmk: (A) A431 cells either left untreated or treated with H35N (8 µg/ml) or zVADfmk (50 µM) for 2 hr followed by addition of H35N (8 µg/ml) for the indicated time which were then mildly trypsinised and viable cells were counted after staining with trypan blue as mentioned in materials and methods. Percentage of stained cells were plotted. The graph represents one of the two independent experiments. (B–D) Cells were treated as mentioned above, stained with PI (2 µg/ml) for 5 min. The samples were analyzed by flow cytometry and atleast 10,000 events were acquired for each sample. (B) control (C) H35N treated cells (D) H35N+ zVADfmk treatment. Apoptotic cell morphology was detected by decrease in forward scatter. (E) Cells post treatment for the following time points were fixed and stained with Hoechst 33342 as mentioned in materials and methods. The lower panel shows merged image of Hoechst staining with phase contrast.

In order to examine whether H35N induced cell death was dependent on caspases, cells were left untreated or treated with the pan caspase inhibitor zVAD-fmk followed by H35N treatment and the cell death was assessed by trypan blue staining. Cell death could not be inhibited by broad spectrum caspase inhibitor, zVADfmk as depicted in Fig. 6A. However, we have also examined the morphology of the H35N induced cell death in presence of zVADfmk by FACS analysis. The decrease in the forward light scatter of cells treated with H35N in presence of zVADfmk indicated that the cell death resembled apoptosis (Fig. 6B-D). In light of this observation, we have also investigated the nuclear morphology of the H35N treated cells in presence of zVADfmk. Interestingly, we observed that there was only partial nuclear condensation in presence of zVADfmk in H35N treated cells as compared to H35N treated cells without zVADfmk, which displayed chromatin condensation along with nuclear shrinkage (Fig. 6E). One possible candidate that mediates partial nuclear condensation without shrinkage is Apoptosis Inducing Factor (AIF) [10], [11]. It is believed that once mitochondria are irreversibly permeabilised, cell death proceeds regardless of caspase activity. AIF is a mitochondrial flavoprotein which localizes in mitochondrial intermembrane space where it performs oxidoreductase function. However, when the mitochondrial membrane gets depolarized, it is released into the cytosol, gets translocated to the nucleus and triggers high molecular weight DNA (50 kb) fragmentation [10], [11]. The mitochondrial-nuclear translocation of AIF was not prevented by broad spectrum caspase inhibitor zVADfmk nor did it prevents its lethal effect indicating that this protein is involved in caspase independent cell death [12]. Furthermore, it has already been shown that the low dose of α-HL which efficiently induce caspase activation in Jurkat T cells, markedly induced high molecular weight DNA breaks along with oligonuleosomal DNA fragmentation [5]. The pan caspase inhibitor zVADfmk inhibited the oligonucleosomal DNA fragmentation but it could not prevent the accumulation of α-HL induced high molecular weight DNA fragments [5]. Hence, in light of these observations and the fact that the H35N induced mitochondrial depolarization leads to the release of mitochondrial proteins, we speculate that H35N also induces translocation of AIF. In summary, these observations suggest that H35N induced cell death is not solely dependent on caspase involvement.

### H35N induces translocation of Phosphatidylserine

**Figure 7:**
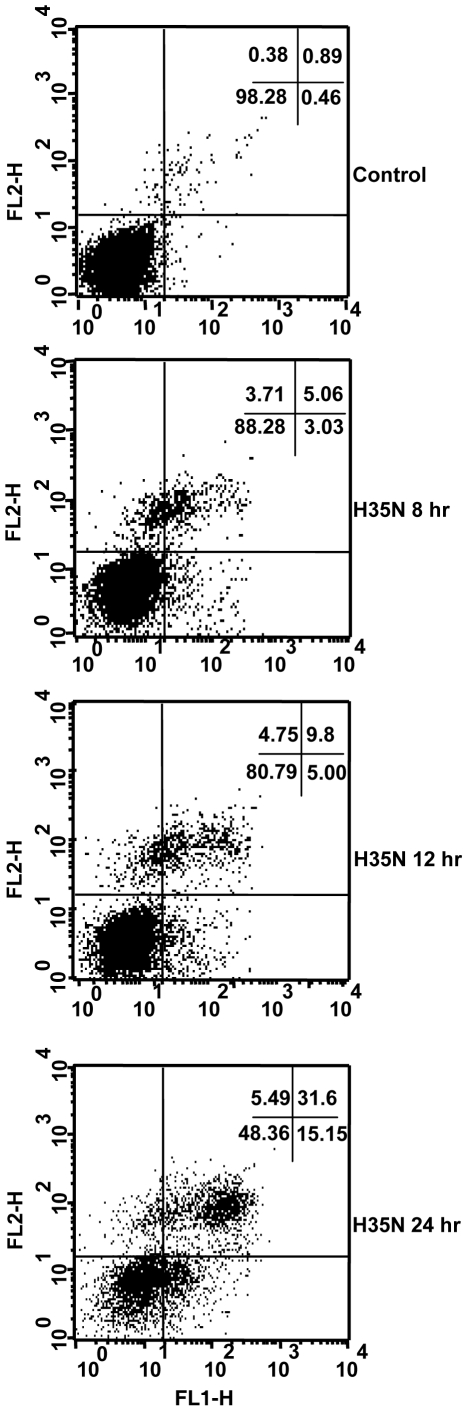
Annexin V and propidium iodide staining of A431 cells upon H35N treatment: A431 cells treated with H35N (8 µg/ml) for the indicated time points followed by double staining with propidium iodide and annexin V-FITC and analysed by flow cytometry as mentioned in materials and methods. FL-1H (green) represents annexin V fluorescence and FL-2H (red) is indicative of propidium iodide staining. One representative experiment of the two is shown.

Phophatidylserine translocation occurs in almost all cell types undergoing apoptosis. Under typical apoptotic conditions, the dying cells first stain positive for annexin V(early apoptotic condition) and then results in an increase in annexin V- propidium iodide double positive population(late apoptotic cell death). Cells treated with H35N for 12 hr or more exhibited an increase in annexin V and propidium iodide uptake but did not produce annexin V single positive cells (Fig. 7). The reason as to why H35N treatment of A431 cells did not result in a dramatic increase in annexin V single positive cells, but resulted predominantly in annexin V-propidium iodide double positive cells was not clear. Although, an identical observation was reported earlier where the caspase activating doses of α-toxin did not result in an increase in population of annexin V single positive cells but resulted in annexin V- propidium iodide double positive cells only [5].

### Membrane integrity is intact in case of H35N but not with α-HL

**Figure 8:**
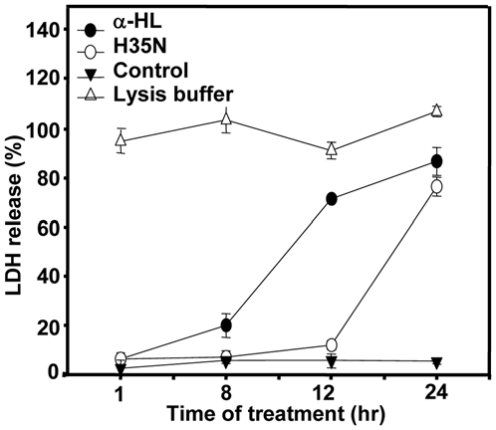
Effect of H35N on LDH release: Cells were incubated with purified H35N or α-HL for the indicated time points and release of LDH in the media was measured using LDH kit. The release of LDH by the cells treated with lysis buffer provided in the kit was considered as 100%. One representative experiment out of the two is shown.

The necrotic form of cell death was also examined by quantitation of LDH release in the extracellular medium, a marker for membrane integrity. In case of H35N treatment, there was no significant release of LDH in the early hours of treatment, i.e., ∼8 hr, but, in ∼24 hr we could see substantial release of this marker, which is expected at later stages of apoptosis (Fig. 8). In contrast, α-HL showed considerable release of LDH in ∼8 hr.

All these observations suggest that the induction of apoptosis by α-HL was due to the presence of the monomeric form of α-HL. Based on the present and published data so far, the reason for caspase activation might be as follows: when α-HL binds to the target cells, only a fraction of the protein converts to lytic pore, while a substantial amount of protein remains as membrane bound monomer and as non-lytic, pre-pore. In the absence of both the pre-pore and the functional pore, our data clearly highlights the activation of the intrinsic apoptotic pathway. Hence, it is possible that the membrane bound monomer activates the intrinsic apoptotic pathway while a successful functional pore assembly results in classical, necrotic form of death. However, the causes for the induction of apoptotic pathway are unclear, as the H35N can neither undergo large scale conformational changes nor it is efficient in inducing membrane damage.

### Link between α-HL binding and caveolae

In our earlier work, we have provided several evidences by biochemical analysis and molecular modeling that the α-HL can interact with the scaffolding domain of Caveolin-1 (Cav-1; amino acids 81–101) through its 9 amino acid binding motif ‘W^179^GPYDRDSW^187^’ [13]. Firstly, α-HL targets itself to the lipid rafts of mammalian cells after binding, as it was detected in Cav-1 enriched membrane fractions and it co-precipitated with Cav-1. Secondly, purified Cav-1 blocked the hemolysis of red blood cells caused by α-HL. Furthermore, treatment of A431 cells with α-HL resulted in clustering of Cav-1 at cell-cell contacts and depletion of cholesterol in A431 cell membranes completely arrested the assembly of α-HL [14], [15]. Theoretically, the Cav-1 matches the dimensions and the stoichiometry of α-HL for a possible, facile assembly of its β-barrel, thereby; Cav-1 can act as an anchor for the α-HL beneath the cell surface through protein-protein interactions. Hence, the presence of Cav-1 at the cell membrane can form a basis for susceptibility or resistance of the target cells towards α-HL.

**Figure 9:**
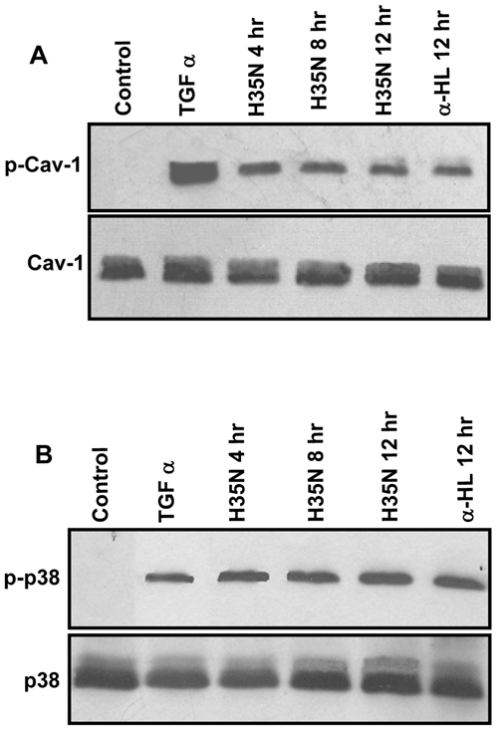
H35N induces phosphorylation of Cav-1 and p-38: (A) Persistent phosphorylation of Cav-1 was detected in cells by immunoblotting following treatment of A431 cells with H35N or α-HL for time points mentioned. TGF-α treatment was taken as positive control. The lower panel represents total Cav-1 as loading control. (B) A431 cells either left untreated or treated with H35N or α-HL for the indicated time points followed by immunoblotting by phospho-p38 antibody. The lower panel shows total p38.

It has been reported in the literature that cell shrinkage was required for tyrosine phosphorylation of Cav-1 and p38 MAP kinase activation [16]. In the light of cell shrinkage observed by H35N treatment, we have examined the changes in the phosphorylation of Cav-1 and p38. Interestingly, we observed that the H35N treatment resulted in the phosphorylation of Cav-1 at tyrosine-14 and p38 MAP kinase as shown in Fig. 9, which may signify stress.

### α-HL and H35N immobilize the dynamic caveolae

**Figure 10:**
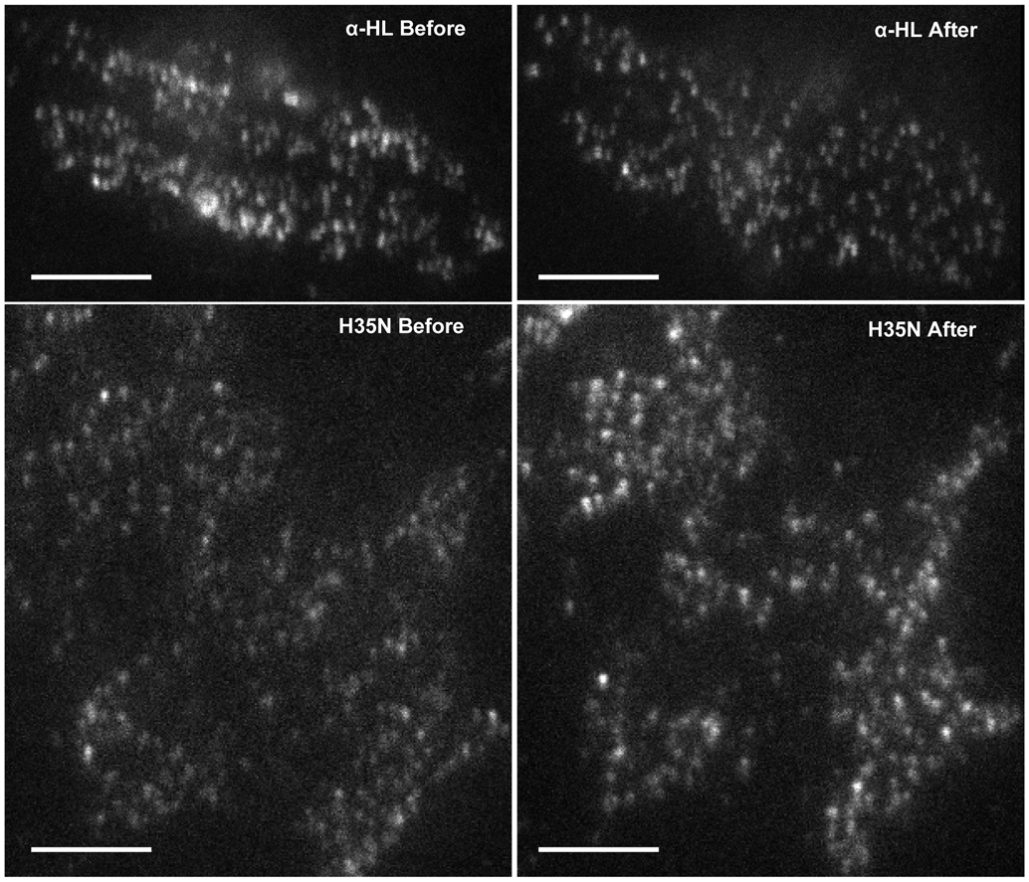
Changes in caveolae dynamics mediated by α-HL and its mutant: (A) HeLa Cav-1-GFP transfected cells were visualized before and after treatment with α-HL (500 ng/ml) for 45 min or H35N (5 µg/ml) for 2 hr using TIRF microscope (100X) oil based objective. One representative frame of each video is shown and the bar represent 10 µm.

**Figure 11:**
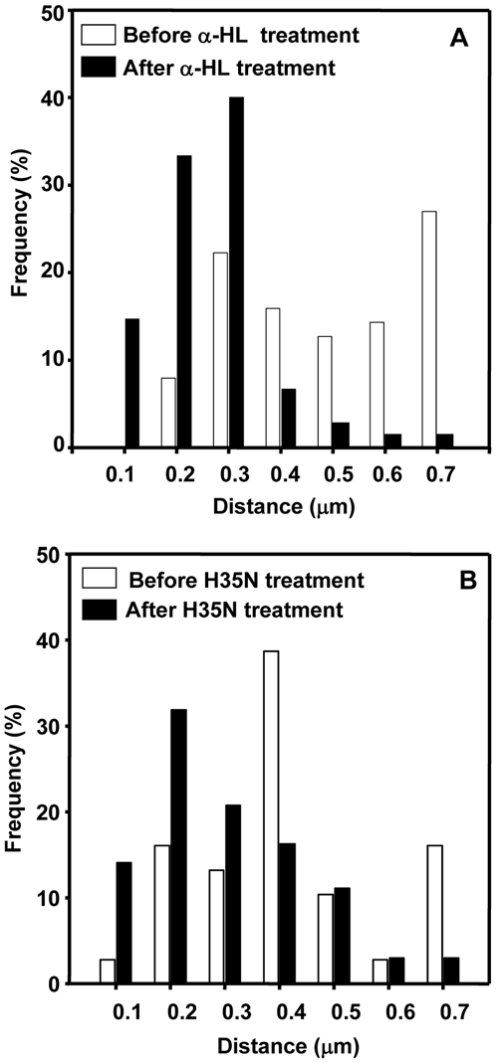
Histogram representation of changes in caveolae dynamics caused by α-HL and its mutant: The panels (A) and (B) respectively represent histograms of movement of the caveolae spots before and after treatment of α-HL and H35N. The X- axis represents the distance in µm and the Y- axis represents the frequency in per cent of caveolae at a given bin value.

The caveolae of mammalian cells exist as static platforms as well as undergo a continuous cycle of ‘kiss and run’ dynamics with the plasma membrane [17]. The kiss and run dynamics of Cav-1-GFP, observed by us in HeLa cells, by Total Internal Reflection Fluorescence Microscopy (TIRFM) was in complete agreement with the motion seen in the videos on HeLa cells shown by Pelkmans and Zerial [17]. While a few caveolae remain static (permanently docked with the plasma membrane), several caveolae undergo fusion and detachment with the plasma membrane within a small volume beneath it (Video S1). In view of our earlier observations which suggested a possible interaction of α-HL with Cav-1, we have examined the dynamics of caveolae since Cav-1 is an important structural component of caveolae. Interestingly, upon α-HL treatment, the kiss and run dynamics was completely arrested at the cell surface within 30 to 45 min (Video S1 vs. Video S2 and Fig. 10) while there was no detectable membrane damage (data not shown). Similar results were also observed with the H35N treatment after 2 hr (Video S3 vs. Video S4 and Fig. 10). The caveolae which normally move ∼735 to 1050 nm^2^ (area wandered by caveolae vesicles was 0.18 to 0.256 µm^2^/sec) remained confined to ∼105 to 315 nm^2^ (area wandered by caveolae vesicles after treatment was 0.025 to 0.076 µm^2^/sec) after α-HL or H35N treatment (Fig. 11A & B). In contrast, W179C, a mutant form of α-HL (which has a mutation in Cav-1 binding motif) did not affect the caveolae dynamics (Video S5). The number of caveolae that remain arrested at the plasma membrane increased by ∼65% upon H35N treatment while we could not observe any increase in the membrane arrested caveolae or change in the nature of motion of caveolae (either slow or rapid movement or internalization) in case of treatment with W179C (Caveolin-1 binding deficient mutant of α-HL). The H35N had an identical effect on A431 cells as the caveolae dynamics were arrested to the similar magnitude (Video S6). It should be noted that the H35N treatment did not result in 100% loss of dynamics like α-HL (Video S2 vs. Video S4) as prolonged incubation with H35N resulted in membrane rounding and cell shrinkage. As a result of these morphological changes, the basal membrane moves away from the cover glass to the limits of evanescent wave excitation range (within ∼200 nm from the cover glass) which is not amenable to TIRF recording. Till date, the significance of kiss and run dynamics of caveolae and the fate of cell when intervened by an artificial means remain largely unknown. In the present study, for the first time we have shown the interference in the caveolae dynamics by an extrinsic protein. Thus, blockade of Cav-1 dynamics appears to be the first event that the target cell recognizes for initiation of appropriate counter measures.

Till date, two serine/threonine kinases (KIAA0999 and MAP3K2) have been shown to regulate the caveolae/raft mediated endocytosis and SV-40 entry [17]. Silencing of these kinases drastically reduced the kiss and run dynamics leading to accumulation of caveolar structures at the cell surface. Probably these kinases may ‘man and sense’ the dynamics of caveolae. The spatio-temporal relationships of these kinases with respect to the caveolae dynamics after H35N treatment are still to be understood and the effector downstream signals generated by the blockade of caveolae dynamics need further investigation.

Our studies are also in agreement with the observations known to date on caveolae kiss and run dynamics [17]. For example, upon exposure of cells to phosphatase inhibitor such as okadaic acid (video S10 in ref [17]), there was an increase in the number of docking events of caveolae at the cell surface. Conversely, activation of phosphatases (shifting of the equilibrium between phosphorylation and dephosphorylation reaction towards dephosphorylation) should result in blockade of caveolae dynamics. In our earlier report, partial assembly of α-HL was shown to elevate the activity of receptor like protein tyrosine phosphatase σ, which belongs to LAR family of protein tyrosine phosphatases [18]. Thus, during the course of α-HL's assembly on the cell surface, the membrane bound monomer can arrest the dynamics of caveolae, while the partially assembled form is responsible for the activation of a phosphatase which might accelerate the blockade of the dynamics of caveolae.

It is evident from the literature that the phosphorylation of Cav-1 plays an important role in cell proliferation, stress and apoptosis [16], [19]. Cav-1 is transiently phosphorylated during growth factor signaling, extracellular stresses such as high osmolarity, H_2_O_2_ and UV light [16], [19]. However, treatment with H35N resulted in persistent phosphorylation of Cav-1 and cell death. Here, an important question arises: Is the persistent phosphorylated form of Cav-1 responsible for the initiation of the intrinsic pathway of apoptosis? Recently, it has been reported that taxol induced apoptosis required Cav-1 phosphorylation and it was found to be persistent [20]. This finding is in line with our observation, wherein, we show that the H35N treatment results in persistent phosphorylation of Cav-1 followed by mitochondrial destablization leading to apoptosis via an intrinsic pathway. Thus, there exists a possible link between the dynamic caveolae, persistently phosphorylated form of Cav-1 and the cell death. In summary, α-HL might have evolved as a smart molecule - which when succeeds in forming functional pore, kills the cell by necrosis and when it fails to proceed beyond membrane bound monomer; it gradually paralyses the cell by apoptosis.

### H35N leads to activation of SREBP-1

**Figure 12:**
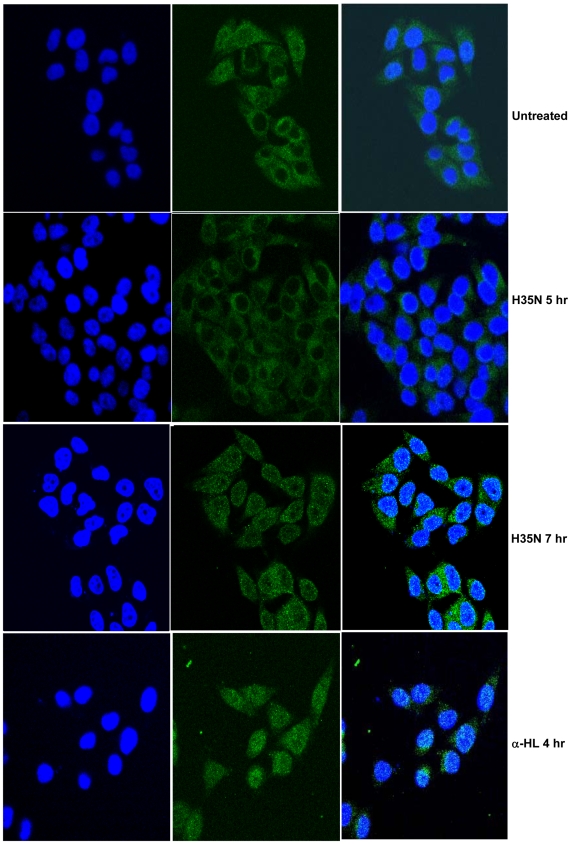
H35N induces translocation of SREBP-1 to the nucleus: HeLa cells were treated with H35N or α-HL for the following time points and stained with SREBP-1 antibody as mentioned in materials and methods. The extreme left panel shows nuclear staining. The middle panel refers to SREBP-1 staining. The extreme right panel represents the merged image of SREBP-1 with DAPI staining.

**Figure 13:**
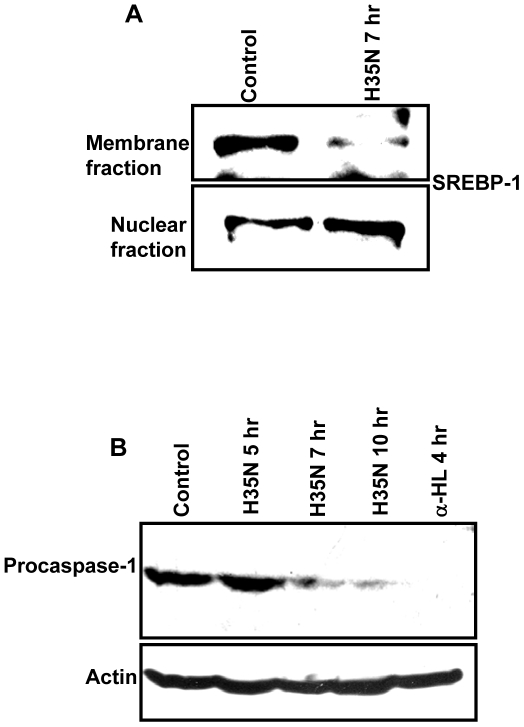
H35N mediated SREBP-1 translocation by immunoblotting and caspase-1 activation: (A) HeLa cells were treated with H35N. Membrane and nuclear fractions were prepared and probed with SREBP-1 antibody. (B) HeLa cells were treated with H35N or α-HL for the following time points followed by immunoblotting with caspase-1 antibody. The lower panel refers to total actin.

The observed blockade of caveolae dynamics by H35N takes place in about 2 hr after binding and the process of cell death was initiated in about 8 hr. It is important to know the event that occurs between 2 hr and 8 hr. The blockade of caveolae dynamics by α-HL and H35N, described above, might be one of the first events that occur after binding of the toxin to the membrane. At this stage, we anticipated either of the two major events to occur: 1. The toxin treated cells may undergo a repair mechanism. 2. And if the repair mechanism is not adequate to mount an effective survival response, the cell death might get initiated. Recently, Gurcel et. al have shown that binding of α-HL on HeLa cells initiated the activation of caspase-1 via the inflammasome pathway by translocation of Sterol Regulatory Element Binding Protein (SREBP) to the nucleus which in turn promotes cell survival upon toxin challenge. SREBPs are membrane bound transcription factors that regulate the expression of genes harboring the sterol reponse elelment (SRE) in their promoter region which are involved in cholesterol and fatty acid biosynthesis. SREBPs reside in the ER and are converted into the mature form by S1P and S2P. Mature SREBPs then migrate to the nucleus and induce the transcription of lipogenic genes which in turn promote cell survival in response to the toxin induced damage of cell membrane. An important point to note from the work presented by Gurcel et. al is that they have used low concentration of the toxin under which all cells excluded propidium iodide for 10 hr. We would like to emphasize here that after the binding of the toxin to the cell surface, only a fraction of the α-HL converts itself to the heptameric form (membrane damageable form) while a substantial portion of the bound α-HL remains as a membrane bound monomer and some as pre-non-lytic pore. Hence, the presence of the membrane bound monomer in their experiments is inevitable. In order to examine the role of the membrane bound monomer's ability to induce membrane damage, we examined the activation and translocation of SREBP-1 protein in HeLa cells post H35N treatment. We observed that after 7 hr of H35N treatment, there is noticeable translocation of the SREBP-1 protein to the nucleus as compared to control (Fig. 12). The translocation of SREBP-1 from ER to the nucleus was further confirmed by immunoblotting. In Untreated cells, full length SREBP-1 was found in the membrane fraction and the amount of full length SREBP-1 decreased upon H35N treatment from the membrane fraction with the appearance of mature SREBP-1 in the nuclear fraction (Fig. 13A).

### H35N triggers activation of caspase-1

SREBP-1 activation is mediated through caspase-1 [21]. Once activated, caspase-1 induces the intermediate targets which then mediate the processing of SREBPs by S1P and S2P. We therefore analysed the effect of H35N on caspase-1. As shown in Fig. 13B, H35N treatment lead to the processing of procaspase-1 with time. Although many details of this pathway still remain to be elucidated, our data indicate that the cells, post H35N treatment, initiated the survival pathway to recover from the H35N induced damage. It must be mentioned here that the notable event that occurred after the binding of H35N was loss of caveolae dynamics (Fig. 10). Hence, it is reasonable to speculate that the loss of caveolae dynamics or dys-regulated dynamics of caveolae was sensed as damage by the host cell.

We believe that the repair response seen in case of HeLa cells was inadequate, hence the reason for initiation of apoptosis by the host cell. Based on the published data so far, it is not clear regarding the initial events and/or the nature of yet to be identified intermediate events that activate the inflammasome pathway. In the present study, we have also treated HeLa cells with α-HL and within 45 min there was a complete blockade of caveolae dynamics. Thus, our work provides valuable insight into the first steps of this pathway. Although, there are several missing links between caveolae trafficking and inflammasome activation.

**Figure 14:**
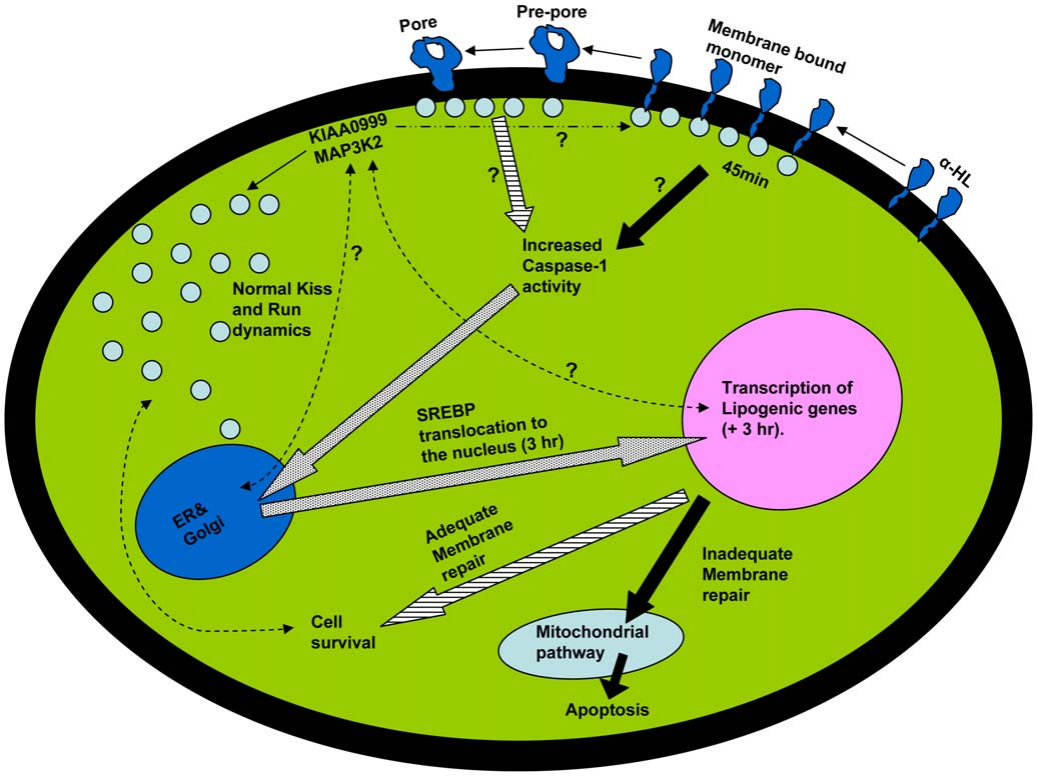
Summary of observations known till date regarding the membrane repair mechanism: When α-HL begins to assemble, the caveolae are arrested at the cell surface within 45 min. The assembly proceeds through irreversible formation of pre-pore and the final transmembrane pore. Once the cell senses the membrane damage, it activates the inflammasome pathway by activating Caspase-1 and processing and translocation of SREBP from ER to Nucleus to initiate the transcription of lipogenic genes. If this attempt is successful, the cell is expected to survive. In case the cell's responses are inadequate, it appears to activate apoptotic death pathway. The big arrows (dash shaded) are the work of Gurcel et al [21]. The Filled big arrows are the information derived from this work. The big arrows (grey dotted) are the overlapping observations of Gurcel et al and this present work. The thin dashed arrows are the pathways that are still to be understood.

In brief, the data available on the cell membrane repair and its consequences have been summarized in the schematic representation shown in Fig. 14. When α-HL binds as membrane bound monomer, the caveolae are arrested at the cell surface within 2 hr. This marks the beginning of stress in the form of Caveolin-1 and p38 phosphorylation. Once the cell senses the stress, it activates the Caspase-1 between 2-7 hr which results in translocation of SREBP-1 from ER to nucleus to initiate the transcription of lipogenic genes. If this attempt is successful, the affected cell might survive. In case, the cell's responses are inadequate, it probably can not restore the caveolae dynamics and eventually the apoptotic death pathway gets initiated after 8 hr via the intrinsic mitochondrial pathway. p38MAP kinase activation is also shown to activate the survival pathway in non immune cells [22]. It is assumed that the pathway observed by us is same as that of the one shown by Gurcel and co-workers since vital events observed by all of us are same. It is not clear about the exact role of KIAA0999 and MAP3K2 kinases (which are involved in controlling the dynamics of caveolae) at this point of time including its link to the inflammasome pathway activation. In summary, it is becoming clear that caveolae trafficking plays a far greater role in the regulation of cell proliferation and death than is currently known.

## Supporting Information

Video S1 TIRFM video of HeLa cells expressing Cav-1-GFP before treatment with α-HL. The video shows normal ‘kiss-and-run’ dynamics of caveolae similar to that of the dynamics reported by Pelkmans and Zerial [1]. The EM gain settings used during the recording was 3420.
(3.37 MB MOV)

Video S2 TIRFM video of HeLa cells expressing Cav-1-GFP after α-HL treatment. The cells in Video S1 were treated with α-HL (500 ng/ml) for 45 min and TIRFM video was recorded as described in methods sections. Note the accumulation of caveolae at the cell surface (bright spots of Cav-1-GFP). The EM gain settings used during the recording was same as video 1.
(3.62 MB MOV)

Video S3 TIRFM video of HeLa cells expressing Cav-1GFP before H35N treatment. The normal kiss-and-run dynamics of caveolae is similar to that of video 1. The EM gain settings used during the recording was 3849.
(7.22 MB MOV)

Video S4 TIRFM video of HeLa cells expressing Cav-1-GFP after H35N treatment. The cells in Video S2 were treated with H35N (5 µg/ml) for 3 hr and TIRFM video was recorded as described in methods sections. The blockade of caveolae dynamics is seen on the cell surface though it is not 100% as in case of α-HL. The EM gain setting was same as video 3.
(6.52 MB MOV)

Video S5 TIRFM video of HeLa cells expressing Cav-1-GFP after W179C treatment. HeLa cells expressing Cav-1-GFP were treated with W179C (5 µg/ml) for 3 hr and TIRFM video recorded as described above. Note that there is no change in the caveolae dynamics. The EM gain settings were 3784.
(5.25 MB MOV)

Video S6 TIRFM video of A431 cells expressing Cav-1-GFP after 3 hr treatment with H35N. The A431 cells expressing Cav-1-GFP were treated with H35N (5 µg/ml) and TIRFM video was recorded as described above. Note the blockade of caveolae dynamics at the cell surface similar to that of video 4. The EM gain settings were 3869.
(6.16 MB MOV)
